# Human Parainfluenza Type 4 Infections, Canada

**DOI:** 10.3201/eid1211.060196

**Published:** 2006-11

**Authors:** Marie-Louise Vachon, Natasha Dionne, Éric Leblanc, Danielle Moisan, Michel G. Bergeron, Guy Boivin

**Affiliations:** * Research Center in Infectious Diseases of the Centre Hospitalier Universitaire de Québec, Quebec City, Quebec, Canada;; † Laval University, Quebec City, Quebec, Canada;; ‡ Centre Hospitalier Régional du Grand-Portage, Rivière-du-Loup, Quebec, Canada

**Keywords:** Human parainfluenza type 4, respiratory diseases, phylogenetic analysis, epidemiology

## Abstract

During the fall/winter season of 2004–05, we found 9 respiratory specimens positive for human parainfluenza virus type 4 (HPIV-4) in our laboratory (43% of all HPIVs) from patients with mild to moderate respiratory illnesses. Sequencing studies identified 8 different HPIV-4A strains and 1 HPIV-4B strain.

Human parainfluenza viruses (HPIVs) have been recognized as a cause of respiratory tract infections for many decades. They belong to the Paramyxoviridae family, subfamily Paramyxovirinae, and are classified into 4 serotypes. Serotype 4 can be further subdivided into 2 antigenic subtypes, HPIV-4A and HPIV-4B (*1*). Although the epidemiology and clinical manifestations of serotypes HPIV-1 to HPIV-3 are well described, much less is known about HPIV-4, including its seasonality (*2*).

HPIV-4 has been mostly associated with mild illnesses (*3**,**4*). However, some evidence has indicated that it can cause more severe infections in some settings (*5**–**10*). We sought to describe the virologic and molecular characteristics as well as the clinical manifestations associated with HPIV-4 infections at our hospital during 2004–05.

## The Study

From October 20, 2004, to March 8, 2005, we found 9 respiratory specimens positive for HPIV-4 in our virology laboratory. Specimens were collected from patients that were either admitted to a tertiary care hospital, seeking treatment at its emergency room, or attending an outpatient clinic connected to that hospital.

Specimens were placed into 96-well plates seeded with 8 cell lines and onto 2 shell vials (Table 1). Viral cultures were incubated for 21 to 24 days. For LLC-MK2 and MDCK cells, a hemadsoption test was performed at the end of the incubation period. Cytopathic effects (CPEs) or positive hemadsorption tests were confirmed by using immunofluorescence assays performed with monoclonal antibodies against HPIV-4 (Chemicon International, Temecula, CA, USA).

**Table 1:** Cell lines used for viral culture and primers used for HPIV-4 PCR testing

Cell lines	Oligonucleotide sequences (5´–3´)	Target genes
Mink lung	ATGGGTGTCAAAGGTTTATC	Fusion
Human foreskin fibroblast	(forward)	
Human lung carcinoma (A-549)		
Vero	AATTATGCAGATTGTAACTGTC	
Hep-2	(reverse)	
Human rhabdomyosarcoma (RD)	ATGGTGAAAAGAACATGGAG	Hemagglutinin-neuraminidase
Transformed human kidney 293	(forward)	
Human colon adenocarcinoma (HT-29)	TGGAGTATCCAGCAGTAAGA	
Madin-Darby canine kidney (MDCK)	(reverse)	
Tertiary monkey kidney (LLC-MK2)		

Viral RNA was extracted from culture supernatants by using the MagaZorb RNA Mini-prep kit (Cortex Biochem, San Leandro, CA, USA) and then tested with RT-PCR by using the Qiagen one-step RT-PCR kit. Amplicons of the fusion (F; 1631 nt) and hemagglutinin-neuraminidase (HN; 1721 nt) genes were generated by using primers listed in Table 1. Nucleotide sequences of all HPIV-4 strains were determined and entered into a multiple alignment generated by the Clustal W software (version 1.83) (*11*). Phylogenetic analyses were performed using distance methods with the PAUP software (version 4.0b10; Sinauer Associates, Sunderland, MA, USA).

Positive HPIV-4 samples consisted of 7 nasopharyngeal aspirates and 2 nasopharyngeal or throat swab specimens. The 9 viruses grew only in LLC-MK2 cells with all but one demonstrating CPEs from 12 to 21 days post-inoculation (mean: 19 days). The CPEs consisted of large and round swollen cells that progressed to destruction of the monolayer, without syncytium formation. The hemadsorption test result was positive for all isolates with subsequent confirmation by immunofluorescence staining. No other virus grew on the other cell lines. Bacterial cultures were done for 4 patients, and 1 culture was positive for Streptococcus pneumoniae.

**Figure 1:**
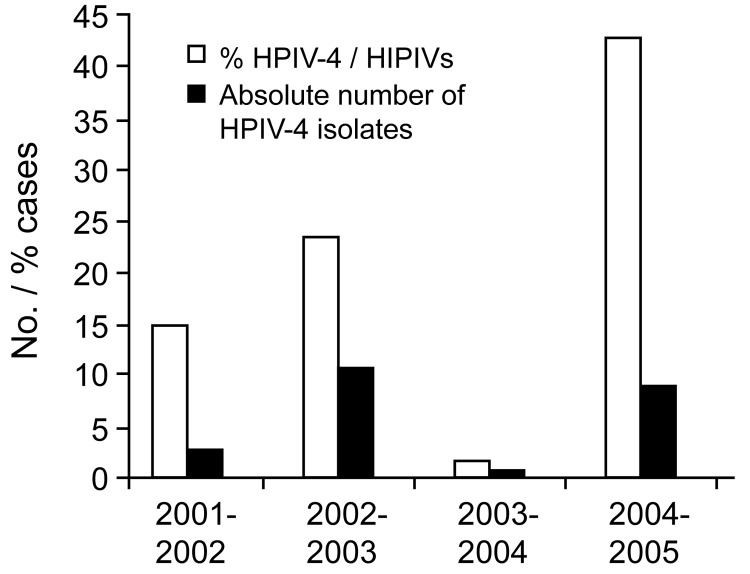
Seasonality of human parainfluenza virus type 4 (HPIV-4) infections during fall and winter of 4 consecutive years.

Between October 20, 2004, and March 8, 2005, 1,424 respiratory specimens were submitted to our virology laboratory for viral culture. Of these, 371 (26%) were positive for a virus. HPIV-4 was the most frequent HPIV with 9 isolates, representing 43% of all HPIVs and 2.4% of all positive cultures. During the same period of 2003–04, we isolated only 1 HPIV-4 (2% of all HPIVs and 0.3% overall). In 2002–03, eleven HPIV-4 were recovered (23% of all HPIVs and 7.0% overall). Finally during the 2001-02 season, we found 3 HPIV-4 (15% of all HPIVs and 0.8% overall) (Figure 1).

The 9 HPIV-4 positive patients consisted of 6 children (5 <6 months of age) and 3 adults (Table 2). Among the pediatric patients, 3 had bronchiolitis, and the youngest (1.5 months) required a stay in the intensive care unit because of apnea. Two of the 3 patients with bronchiolitis had paroxysmal cough, and pertussis was suspected. The other 3 patients had upper respiratory tract illnesses. Among those 6 patients, 2 received antimicrobial drugs, and all recovered.

**Table 2:** Clinical data of 9 patients with HPIV-4 infections in Quebec City, Canada*

Patient	Sample type (date of collection)	Age/sex	Underlying Illness	Symptoms and signs	Hospital-ization (d)	Final diagnosis
1	NPA (2004 Dec 5)	1.5 mo/M	–	Cough, apnea, fever, low O_2_ sat.	Yes, ICU (3)	Bronchiolitis
2	NPA (2004 Oct 20)	2.5 mo/M	Premature (32 wk), POF, PPS	Cough, apnea, low O_2_ sat.	Yes (14)	Bronchiolitis
3	NPA (2004 Nov 2)	3 mo/M	–	Cough, apnea, BOM	Yes (2)	URTI and BOM
4	NPA (2005 Jan 4)	5 mo/M	Premature (33 wk)	Cough, fever	No	URTI
5	NPA (2005 Jan 19)	6 mo/F	Premature (26 wk), PD	Rhinorrhea, wheezing	No	Bronchiolitis
6	NPA (2004 Nov 18)	2.7 y/F	Asthma	Rhinorrhea, cough, wheezing	No	Sinusitis and bronchospasm
7	TS (2005 Mar 8)	25 y/M	–	Fever, right tonsil ulcerative lesions	No	Viral pharyngitis
8	NPA (2005 Jan 25)	84 y/F	CHD, COPD	Dyspnea, low O_2_ sat., cyanosis	Yes (14)	PE, MI, and persisting bronchospasm
9	NPS (2005 Jan 21)	90 y/F	AF, dementia	Fever, muscle aches	No	Flulike syndrome

*HPIV-4, human parainfluenza virus type 4; NPA, nasopharyngeal aspirate; O_2_ sat., oxygen saturation; ICU, intensive care unit; POF, permeable oval foramen; PPS, peripheral pulmonary stenosis; BOM, bilateral otitis media; URTI, upper respiratory tract infection; PD, pulmonary dysplasia; TS, throat swab; CHD, coronary heart disease; COPD, chronic obstructive pulmonary disease; PE, pulmonary edema; MI, myocardial infarction; NPS, nasopharyngeal swab; AF, atrial fibrillation.

Among the 3 adults, 1 was 25 years of age, and 2 were >80 years of age. The former had pharyngitis and buccal ulcerative lesions. No other viral or bacterial pathogen was found. An 84-year-old woman was admitted to the hospital for severe bronchospasm and suspected pulmonary edema. In addition, a 90-year-old woman had a flulike illness but antigenic test results were negative for influenza. The 3 patients had no complications and survived. Overall, 4 of the 9 patients were hospitalized with a mean length of stay of 8 days (range 2–14 days). Six (66%) of the patients had an underlying disease.

**Figure 2:**
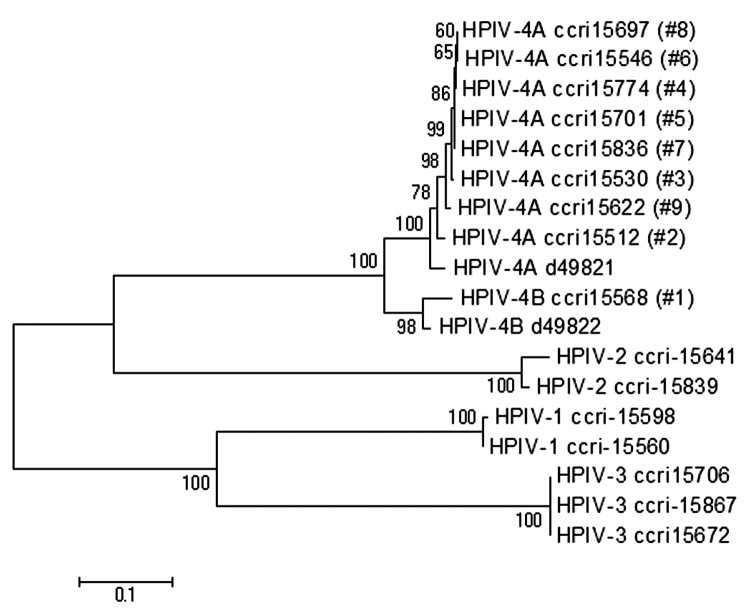
Phylogenetic analysis based on fusion (F) gene nucleotide sequences from clinical and reference human parainfluenza virus (HPIV) strains. The tree was built by using distance method and the neighbor-joining algorithm with Kimura 2 parameters. The topologic accuracy of the tree was evaluated by using 500 bootstrap replicates. Strains isolated at the Research Center in Infectious Diseases (Quebec, Canada) are indicated by a specific identification number (ccri) followed by the patient number in parentheses. Public sequences for HPIV-4A (GenBank accession no. d49821) and HPIV-4B (GenBank accession no. d49822) strains are also indicated in the tree.

Phylogenetic trees of the F and HN genes were similar. Eight different HPIV-4 isolates clustered with the HPIV-4A reference strain, whereas the remaining one clustered with the HPIV-4B reference strain (Figure 2). The percentages of nucleotide (nt)/amino acid (aa) identity for the 8 HPIV-4A strains were 97%/100% (F gene) and 97%/99% (HN gene). In contrast, the percentages of nt/aa identity between the HPIV-4A strains and the HPIV-4B isolate were 89%/92% (F gene) and 86%/87% (HN gene).

## Conclusions

HPIV-4 is considered a rare pathogen because it has been isolated only occasionally from respiratory tract specimens, although seroprevalence studies have shown that 70%–90% of young adults have specific antibodies against it (*12**,**13*). Lately, a few reports have shown that HPIV-4 might be more frequent than previously thought when sensitive RT-PCR methods are used (*5**,**8**,**10**,**14*). Using viral cultures, we found that HPIV-4 accounted for 43% of all HPIVs isolated in our laboratory during the 2004-05 fall and winter seasons. Direct testing of clinical specimens with RT-PCR would have likely resulted in higher detection rates.

Possible explanations for the rarity of HPIV-4 are in part related to its slow growth in LLC-MK2 cells, a cell line not used in most virology laboratories. Also, CPEs are not always present (may take 2–3 weeks to appear), and the hemadsorption reaction is occasionally weak (*1*). Finally, symptoms associated with HPIV-4 are generally mild and do not elicit requests for a cell culture.

To our knowledge, no seasonality has been described for HPIV-4. In our area, HPIV-4 was isolated every year during the last 4 years with peaks of activity occurring every other year, similar to HPIV-1 and 2 (*15*). The biennial pattern of HPIV-4 would require confirmation in larger studies from other countries. In temperate countries, the virus is usually recovered during the late fall and winter seasons (*2**,**13**,**14*). In fact, over the last 4 years, only 4 HPIV-4 isolates were recovered outside our study period, 3 in April and 1 in May.

The retrospective aspect of our study and the small number of viral isolates limit definitive conclusions on the clinical manifestations of HPIV-4 infections. We note that young children were preferentially affected as previously reported (*2**,**5**–**7**,**14*) although they also constitute the most likely population for whom viral cultures would be obtained. In children, clinical conditions included upper respiratory tract infections, bronchiolitis, and pertussislike clinical syndromes (*7*). Infected adults had various clinical presentations, i.e., pharyngitis, bronchospasm, and flulike illnesses, although further work is required to describe the full clinical spectrum of HPIV-4 infections.

Our 9 HPIV-4 isolates could be further subdivided into 8 different HPIV-4A and 1 HPIV-4B strains, according to sequences obtained from the 2 glycoproteins F and HN. Reports of HPIV-4B infection have been infrequent in the last 2 decades, similar to our findings (*9*).

In summary, HPIV-4 infections can be relatively common during the fall and winter seasons of some years and are probably underdiagnosed due to their fastidious growth. Detection of this respiratory pathogen needs to be improved through rapid molecular assays.
